# Electrophoretic deposition of hydroxyapatite–capsaicin nanocomposite coatings on Mg–Ag alloy: effects on microstructure, hardness, and proliferation/chondrogenic differentiation of adipose-derived stem cells

**DOI:** 10.3389/fbioe.2026.1851739

**Published:** 2026-08-26

**Authors:** Xinyi Shi, Taihua Wang, Jianjun Qiao

**Affiliations:** 1 Tianjin University, Guangdong Cell Biotechnology Co., LTD., Dongguan, Guangdong, China; 2 Guangdong Cell Biotechnology Co., LTD., Shenzhen Taihua Cell Biotechnology Co., LTD., Dongguan, Guangdong, China; 3 College of Chemical Engineering, Tianjin University, Tianjin, China

**Keywords:** adipose-derived stem cell, chondrogenic differentiation, composite, deposition, hydroxyapatite

## Abstract

This paper explores the fabrication of hydroxyapatite–capsaicin nanocomposite coatings on Mg–Ag alloy by the electrophoretic deposition (EPD) method at different deposition times and voltages. The microstructure, surface chemistry, hardness, corrosion behavior, and cell proliferation of the deposited layers at different deposition times and voltages were examined. Based on microstructural observations, the deposited layers became denser at higher deposition time and voltage. In addition, the hydroxyapatite–capsaicin nanocomposite became thicker at higher EPD voltage. FTIR and XPS analyses confirmed the successful incorporation of capsaicin into the HAP coating with partial retention of CAP-derived surface functionalities after annealing. Also, the hardness of the coated layer increased with voltage and time. However, EPD voltage was more effective in increasing the hardness. The maximum hardness of 141 HV was obtained at a deposition voltage of 160 V. The hardness profile of the deposited composite showed greater fluctuations with increasing deposition voltage. Electrochemical polarization tests demonstrated that corrosion resistance improved with increasing deposition voltage and time. Furthermore, cell growth was improved at higher EPD time and voltage. MTT assay results confirmed the enhanced cytocompatibility of the HAP–CAP coatings. Also, the Mg^2+^ ion release rate and pH variation decreased as the deposited layer became thicker and denser. The minimum Mg^2+^ ion release rate of 5.2 ppm h^-1^ was obtained at a deposition voltage of 160 V. Compared with conventional HAP coatings, incorporation of capsaicin enhanced the biological performance while preserving the structural stability of hydroxyapatite, which thus provides a promising strategy for the development of multifunctional biodegradable implant coatings.

## 1 Introduction

Materials are continuously being developed to meet the growing needs of many industries and technologies including biomedical engineering, aerospace, automotive, energy, electronics, and environmental sectors (Iskandar et al., 2013; Jo et al., 2011; Supri et al., 2025; Fauz et al., 2025; Widekdo et al., 2025). Advanced materials are expected to outperform conventional materials (Astuti et al., 2025; Siahaan et al., 2025; Rumhayati and Fardiyah, 2025; Setyawan et al., 2025; Ponidi et al., 2024). They should offer higher strength, electrical conductivity, corrosion resistance, biocompatibility, and adjustable surface properties (Puspitasari et al., 2025; Maulana et al., 2025; Nabila et al., 2025; Anggayasti et al., 2025; Meizarini et al., 2025). As a result, composites have become an interesting option because they combine the advantages of their substances (Kartikowati et al., 2025; Baihaqi et al., 2025; Nagaraju et al., 2025; Juliananda et al., 2025; Hayati et al., 2025). In the biomedical field, various strategies are used to improve implant performance. These strategies involve modifying the bulk material or changing its surface (Aisyah et al., 2025; Erwin and Sugiarto, 2025; Ma et al., 2025). Surface modification has received much attention because the body’s response to an implant depends largely on interactions at the material–tissue interface (Yanuhar et al., 2025; Nurlailiyah et al., 2025; Wargasetia et al., 2025). As a result, surface engineering methods have received considerable attention. Among them, coating is one of the most effective techniques for enhancing surface properties without harming the bulk material’s mechanical strength. Bioactive coatings in recent years have been developed to improve the clinical performance of biomedical implants through enhancing corrosion resistance, cell attachment, and tissue growth (Lv et al., 2023; Assidqi et al., 2025; Annisa et al., 2025).

Magnesium alloys are widely utilized in medical implants because of their high strength, low density, and non-toxicity potential (Li et al., 2017). Even though biocompatible Mg alloys are largely applicable in orthopedic applications, they usually suffer from sudden degradation after implantation. To overcome this problem, alloying elements and coatings are often used. Among various alloying elements, Ag has attracted considerable attention because of its antibacterial properties (Liu Z. et al., 2017; Choirunnisa et al., 2025). The antibacterial effect of Ag on Mg-Ag alloys has been studied in recent years. The coating methods are widely employed to improve the hardness, wear performance, corrosion resistance, and biological features (Thakur et al., 2022). Various coating biomaterials have been used for Mg alloys to enhance bone-cell bonding, cell adhesion, and bone growth (Iskandar et al., 2013; Iskandar et al., 2015). However, achieving simultaneous corrosion protection and enhanced biological performance remains a major challenge for biodegradable Mg implants.

For the selection of coating material, the bioactivity and chemical bonding to bone should be taken into account (Hao et al., 2024). To enhance cell adhesion, cell proliferation, tissue repair, and bone regeneration, Hydroxyapatite (HAP) is one of the bioactive materials with similar properties to human bone (Bal et al., 2020). For instance, nano-hydroxyapatite-based scaffolds have been recently used in bone tissue engineering due to their excellent osteoconductive and biocompatible characteristics (Rachmawa et al., 2025). In the past decades, various composites containing HAP have been fabricated (Rapacz-Kmita et al., 2006; Khanra et al., 2010). Having similar characteristics to human bone, HAP is extensively utilized in medical applications. HAP coatings have been widely applied to metallic biomaterials to improve their biological performance (Amirtharaj Mosas et al., 2022). The metallic substrate provides the required mechanical strength, whereas the coating provides bioactivity. Owing to its similar composition to human bone, HAP is attracting much attention in tissue engineering. Because of its excellent bioactivity and biocompatibility, HAP is widely used in the medical field. However, it suffers from poor mechanical properties that limit its applications (Veljović et al., 2011). Very recently, HAP was coated on Mg alloy, and cell proliferation on coated surfaces was studied (Hiromoto and Yamazaki, 2017). It has been reported that superior biocompatibility was obtained. The incorporation of HAP onto Mg alloy surfaces helps bone generation. When the ions of phosphate and calcium are dissolved, the carbonated apatite is precipitated which enhances cell adhesion and absorption of protein (Mardiyantoro et al., 2025; Wu et al., 2025; Wang et al., 2024; Setiawan et al., 2025; Maharani et al., 2025). HAP is often combined with other materials as composite materials and coating materials to assist cell adhesion and bone generation (da Silva et al., 2024). Although HAP coatings effectively improve corrosion resistance and osteoconductivity, they exhibit limited intrinsic antibacterial and anti-inflammatory activity. Therefore, incorporating biologically active molecules into HAP coatings has emerged as a promising strategy for developing multifunctional implant surfaces capable of simultaneously regulating degradation and enhancing tissue regeneration.

Owing to its anti-inflammatory, antibacterial, and antioxidant features, Capsaicin (C_18_H_27_NO_3_) has attracted much attention in biomedical materials (Wang et al., 2022). According to the literature (Padmavathi et al., 2025; Truong and Jones, 2018), the addition of capsaicin to biomaterials is beneficial because it hinders the outgrowth of bacteria and promotes cellular responses during tissue repair. In contrast to polymeric or ceramic additives that are used to improve mechanical properties, capsaicin positively affects biological activity (Wang et al., 2022). In recent years, capsaicin has been used in various biomaterials to improve antibacterial activity, modulate inflammatory responses, and promote tissue regeneration. For example, (Padmavathi et al., 2025) studied the addition of capsaicin to biocompatible silicone polymer which led to improved antibacterial activity. In another investigation, Truong and Jones (Truong and Jones, 2018) reported a reduced inflammatory response in capsaicin-embedded implants. However, the incorporation of capsaicin into hydroxyapatite coatings deposited on biodegradable magnesium alloys has not received attention yet. In addition to its well-documented antibacterial and anti-inflammatory properties, capsaicin may contribute to a more favorable biological microenvironment by reducing inflammatory responses around the implant and supporting cellular activity during tissue repair. Therefore, combining capsaicin with HAP is expected to complement the osteoconductive function of HAP while providing additional biological functionality that cannot be achieved using HAP alone.

For orthopedic and dental utilization, hydroxyapatite composites have emerged. Fabrication of nanocomposites based on HAP has received much attention due to the antimicrobial features of HAP. Various materials including metals, ceramics, and polymers can be combined with HAP to fabricate composites with enhanced properties (Rapacz-Kmita et al., 2006; Khanra et al., 2010; Huixia et al., 2016). In recent years, reinforcement in the form of fibers, whiskers, and particles has been employed in HAP composites. In recent years, several investigations about biocompatibility response of HAP-based nanocomposites have been performed. For instance, tungsten oxide-HAP nanocomposite showed no cytotoxicity response and good antibacterial properties (Silingardi et al., 2022). In another investigation, the titania-HAP nanocomposite showed low antibacterial rates (Mohseni-Salehi et al., 2018).

Different coating techniques have been used for nanocomposite coatings on metallic materials. Although these coating techniques have been widely applied to Ti-based alloys and stainless steels, relatively limited information is available regarding their application to Mg–Ag alloys. For instance, recent work by Zhang et al. (Zhang et al., 2022) utilized plasma electrolytic oxidation on an Mg-Ag alloy. They reported a satisfying balance between its corrosion resistance and antimicrobial capability. Similarly, electrochemical deposition has been employed by Singh et al. (Singh et al., 2025) who employed dip coating and spray coating to produce nanocomposite coatings of superhydrophobic coating on Mg-Nd-Zn-Zr alloys which resulted in improved cytocompatibility and corrosion resistance. Nevertheless, limited reports have addressed the fabrication of nanocomposite coatings on Mg-Ag-Zn-Ca alloys. For instance, Lv et al. (Lv et al., 2023) fabricated PEO/Mg–Mn LDH composite coating on an Mg–Ag–Mn alloy which led to corrosion resistance and antibacterial properties. In addition, Mohammadi-Zerankeshi et al. (Mohammadi-Zerankeshi et al., 2023) synthesized a hydroxide coating on an Mg-2Ag alloy that led to enhanced corrosion performance. Therefore, limited insight into the effects of nanocomposite coatings on the biological performance of Mg-Ag alloys has been reported. Additionally, Saremi et al. (Saremi et al., 2014) employed the electrodeposition technique to deposit a nano-hydroxyapatite coating layer on AZ31 alloy. Among various coating techniques, electrophoretic deposition (EPD) is an interesting method to manufacture nanocomposite coatings. This is mainly because coating thickness and microstructure can be effectively controlled by adjusting the EPD processing parameters. Such flexibility makes EPD particularly suitable for fabricating multifunctional composite coatings on biodegradable magnesium alloys.

In orthopedic applications, cartilage defects are the main challenge (Liu Y. et al., 2017). Tissue engineering has employed new methods in solving this problem by using adipose-derived stem cells (ADSCs) (Sterodimas et al., 2010; Wang et al., 2025). Owing to their high proliferation capacity, ADSCs have shown great potential in bone regeneration (Qin et al., 2023). They can be differentiated into various cell lineages such as adipogenic, osteogenic, myogenic, and chondrogenic (Rada et al., 2011). The osteogenic differentiation of the Mg-scaffold has been studied by Wang et al. (Wang et al., 2021). In another study, chondrogenic differentiation of ADSC on Mg and Mg alloy was examined (Sanchez et al., 2017).

Although HAP coatings have been extensively investigated for biodegradable magnesium alloys, studies on HAP–capsaicin nanocomposite coatings deposited on Mg–Ag alloys are scarce. In addition, there is limited information on the use of capsaicin as a bioactive component in HAP-based coatings for biodegradable Mg alloys. Here, capsaicin, as a bioactive component, was incorporated to elevate the biological activity of HAP-coated Mg–Ag alloys that are widely used in bone-regeneration applications. In addition, no systematic study has taken into account the effect of electrophoretic deposition (EPD) parameters on the microstructure, hardness, and biological response of HAP–CAP coatings deposited on Mg–Ag alloys. The current investigation is expected to provide new insight into the design of multifunctional biodegradable coatings by demonstrating how capsaicin incorporation together with optimization of EPD parameters influences coating microstructure, corrosion behavior, and stem-cell response. These findings may contribute to the development of bioactive Mg-based implants with improved biomedical characteristics.

Therefore, the objective of this study was to develop HAP–CAP nanocomposite coatings on Mg–Ag alloy using electrophoretic deposition. Also, the influence of deposition voltage and deposition time on coating morphology, phase constitution, hardness, Mg ion release, and proliferation-chondrogenic differentiation of adipose-derived stem cell response were evaluated.

## 2 Materials and methods

### 2.1 Materials and preparation

In this research, raw materials including nano-sized hydroxyapatite (HAP, Ca_10_ (PO_4_)_6_(OH)_2_) and capsaicin (CAP, C_18_H_27_NO_3_) supplied from Meruax Company were used. HAP was chosen since it possesses excellent biocompatibility and chemical similarity to the mineral phase of natural bone (Liu et al., 2024; Heimann and Lehmann, 2015). Capsaicin was selected as a bioactive additive because of its antibacterial, anti-inflammatory, and antioxidant features, which may contribute to improving the biological performance of implant coatings (Truong and Jones, 2018; Jayan et al., 2025). The combination of HAP and CAP was developed here to develop multifunctional coatings for Mg-based implants.

To clean the substrate surface before the coating process, Mg–Ag alloy plates were surface-treated in consecutive steps including grinding with SiC abrasive papers, cleaning in acetone, rinsing with distilled water, etching in a H_2_O_2_ + H_2_SO_4_ solution, and final washing with distilled water.

### 2.2 Electrophoretic deposition process (EDP)

To prepare the suspension of HAP–CAP, 2.8 g of nano-sized HAP and 0.7 g of CAP were first mixed with 20 mL of distilled water, which was followed by the addition of 60 mL of ethanol. Next, to ensure homogeneous dispersion of the particles before performing the EDP process, the suspension (water: ethanol = 1:3 v/v) was magnetically stirred for 120 min. Therefore, the suspension was prepared in the absence of the substrate to minimize any possibility of premature corrosion.

**FIGURE 1:**
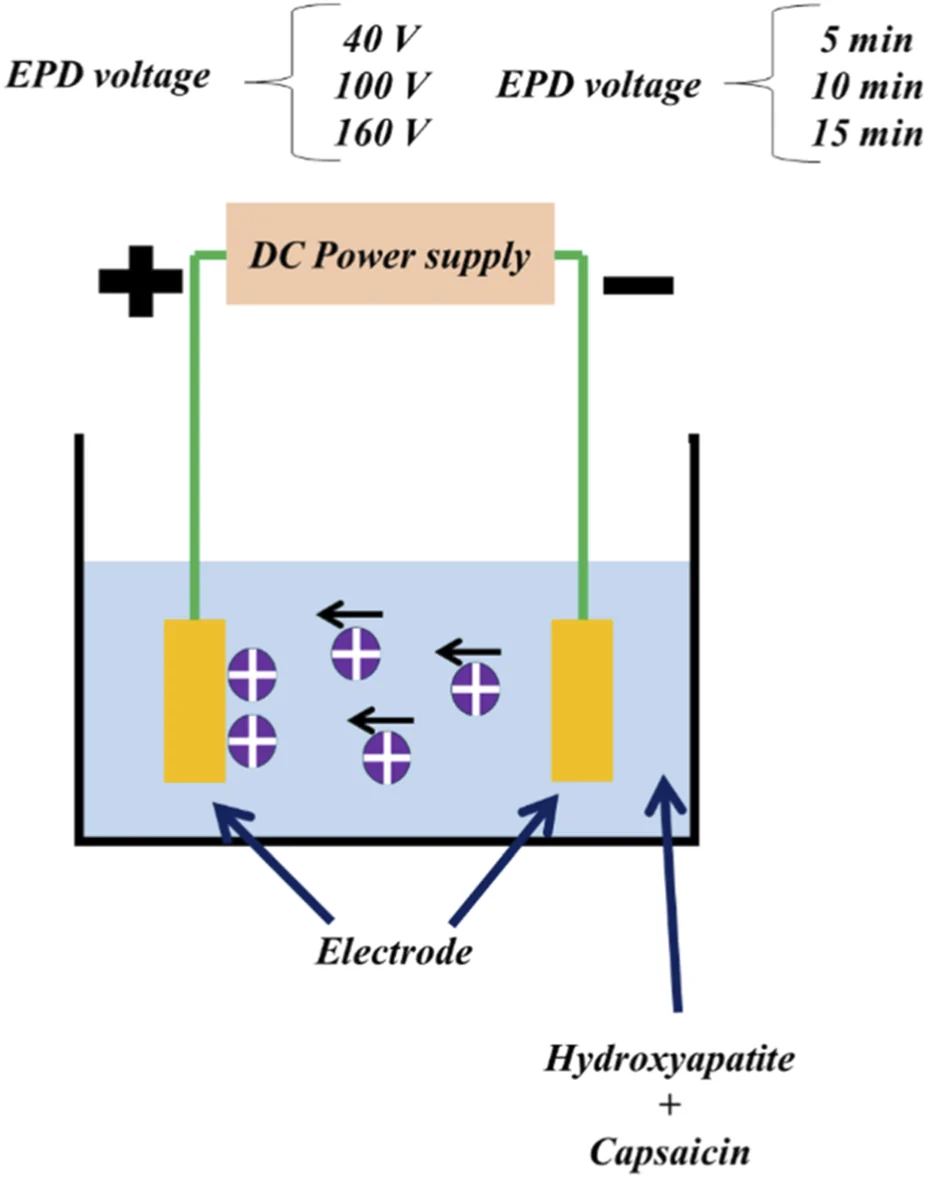
Schematic of electrophoretic deposition method.

Then, the EPD was carried out at ambient temperature, voltages of 40, 100, and 160 V, and deposition times of 5, 10, and 15 min. The anode and cathode were platinum and Mg-Ag alloy plates, respectively. The distance between the anodic and cathodic plates was 20 mm. The Mg–Ag alloy specimens had dimensions of 20 × 20 × 5 mm^3^. The exposed surface area used for electrophoretic deposition was 20 × 20 mm^2^. Table 1 lists the current densities at different deposition voltages and times. After applying voltage, the charged particles were deposited on the anodic and cathodic electrodes. Once the deposition was done, the samples were held at ambient temperature for 1 h to complete the aging process. Next, the samples were dried at 318 K for a dwell time of 60 min. Finally, the dried samples were annealed at 773 K for 120 min with a 2 K/min rate of heating/cooling on the coating layers. The schematic illustration of the EPD method is presented in Figure 1.

**TABLE 1:** Average current density measured under different EPD voltages and times.

*Voltage (V)*	*Deposition time (min)*	*Average current density (mA/cm* ^*2*^ *)*
*40* *40* *40*	5 10 15	1.2 1 0.8
*100* *100* *100*	5 10 15	4.5 3.8 3.2
*160* *160* *160*	5 10 15	10.5 8.8 7.2

To measure zeta potential of the HAP and HAP–CAP suspensions, a Zetasizer Nano ZS (Malvern Panalytical, UK) was utilized which was based on the electrophoretic light scattering (ELS) technique at 25° C. Prior to measurement, to ensure homogeneous particle dispersion, the suspensions were ultrasonically dispersed for 10 min. The average of three measurements for each suspension was reported.

### 2.3 Microstructural characterization

To characterize the microstructures of the nanocomposites, a scanning electron microscope (SEM, VEGA3, TESCAN, Czech Republic) with an accelerating voltage of 15 kV was used. In addition, an X-ray diffractometer (D8 Advance, Bruker, Germany) with Cu Kα radiation (λ = 1.5406 Å) was used at 40 kV and 40 mA to detect phases. To this end, the diffraction patterns were recorded over a diffraction angle range of 20°–80° with a step size of 0.02° and a scanning rate of 2° min^-1^.

To investigate the chemical composition of the nanocomposites and the presence of capsaicin after annealing, Fourier transform infrared (FTIR) spectroscopy was carried out. FTIR spectra of the HAP coating and HAP–CAP coating before and after annealing, were recorded using a Bruker Tensor 27 FTIR spectrometer (Bruker, Germany) in the wavenumber range of 4000–400 cm^-1^ with a spectral resolution of 4 cm^-1^. For each measurement, 32 accumulated scans were done.

The surface chemical composition and chemical states of the annealed HAP and HAP–CAP coatings were evaluated *via* X-ray photoelectron spectroscopy (XPS-Thermo Scientific K-Alpha, USA) equipped with a monochromatic Al Kα X-ray source (hν = 1,486.6 eV). Survey spectra were obtained over a binding energy range of 0–1,200 eV. Also, high-resolution spectra of the C 1s, N 1s, O 1s, P 2p, and Ca 2p regions were obtained. All binding energies were referenced to the adventitious C 1s peak at 284.8 eV.

The surface roughness of the deposited layers was measured *via* a surface roughness tester (SJ-210, Mitutoyo, Japan). The average roughness (Ra) values of five measurements done at different locations on each sample were reported. In addition, the hardness of the deposited nanocomposites was measured using a Vickers hardness tester (HMV-G21, Shimadzu, Japan) using a load of 5 N and a dwell time of 10 s. The average values of five indentations performed on different regions of each sample were reported.

### 2.4 Corrosion, Mg ion release, and pH measurements

The corrosion behavior of the deposited layers was evaluated by performing immersion and electrochemical tests in simulated body fluid (SBF). For these tests, the SBF solution was prepared based on Kokubo’s protocol. Also, the solution temperature was maintained at around 37 °C. Next, each sample was immersed in 100 mL of SBF using a solution volume-to-surface area ratio of approximately 20 mL cm^-2^. Then, immersion tests were conducted for 1, 3, and 7 days. The pH of the SBF was measured *via* a calibrated digital pH meter before immersion and after 1, 3, and 7 days of immersion. Furthermore, after immersion, the solutions were collected and analyzed by inductively coupled plasma optical emission spectroscopy (ICP-OES, Optima 8,000, PerkinElmer, USA) to measure the concentration of released Mg ions. The Mg ion release rate was calculated from the measured Mg concentration and immersion duration. The average values of three independent measurements for each condition were reported. Electrochemical measurements were carried out using a potentiostat (Autolab PGSTAT302N, Metrohm, Netherlands) in SBF at a temperature of 37 °C. For these tests, a conventional three-electrode cell consisting of the sample as the working electrode, a saturated calomel electrode as the reference electrode, and a platinum sheet as the counter electrode was employed. Before polarization testing, the samples were immersed in SBF for 30 min to stabilize the open-circuit potential (OCP). Potentiodynamic polarization tests were carried out in a potential range of −250 mV to +250 mV relative to the OCP at a scan rate of 1 mV s^-1^.

### 2.5 Cell culture and biological evaluation

Adipose-derived stem cells (ADSCs) were purchased from Cyagen Biosciences (Guangzhou, China). According to the supplier, the ADSCs were isolated from human subcutaneous adipose tissue obtained from surgical specimens under approved ethical procedures with informed donor consent. The cells were cultured in Dulbecco’s Modified Eagle Medium (DMEM) supplemented with 10% fetal bovine serum (FBS), 100 U mL^-1^ penicillin, and 100 μg mL^-1^ streptomycin under standard culture conditions including a temperature of 37 °C and a humidified atmosphere containing 5% CO_2_. Before cell seeding, the surfaces of samples including Mg substrate, HAP-coated, and HAP–CAP-coated, were sterilized by immersion in 70% ethanol at room temperature, which was followed by rinsing with warm phosphate-buffered saline (PBS). ADSCs were seeded onto the samples in 48-well plates at a density of 1 × 10^4^ cells per well and incubated at 37 °C in a humidified atmosphere containing 5% CO_2_. For chondrogenic culture, the medium consisted of DMEM supplemented with 10% fetal bovine serum (FBS), 100 U mL^-1^ penicillin, 100 μg mL^-1^ streptomycin, 10 ng mL^-1^ transforming growth factor-β1 (TGF-β1), 50 μg mL^-1^ ascorbic acid, and 1% insulin-transferrin-selenium (ITS). ADSCs were cultured in the chondrogenic medium for 7 days. The cell attachment and morphology were examined by SEM.

Cytocompatibility was quantitatively evaluated using an MTT assay. ADSCs were cultured on the sample surfaces for 72 h. After that, the culture medium was replaced with fresh medium containing MTT reagent (0.5 mg mL^-1^). Following incubation for 4 h, the resulting formazan crystals were dissolved in dimethyl sulfoxide (DMSO). Finally, the absorbance was measured at 570 nm using a microplate reader. In addition, cell viability was calculated based on three independent experiments relative to the control group including uncoated Mg-Ag alloy and HAP-coated Mg-Ag alloy.

## 3 Results and discussion

### 3.1 Morphology

**FIGURE 2:**
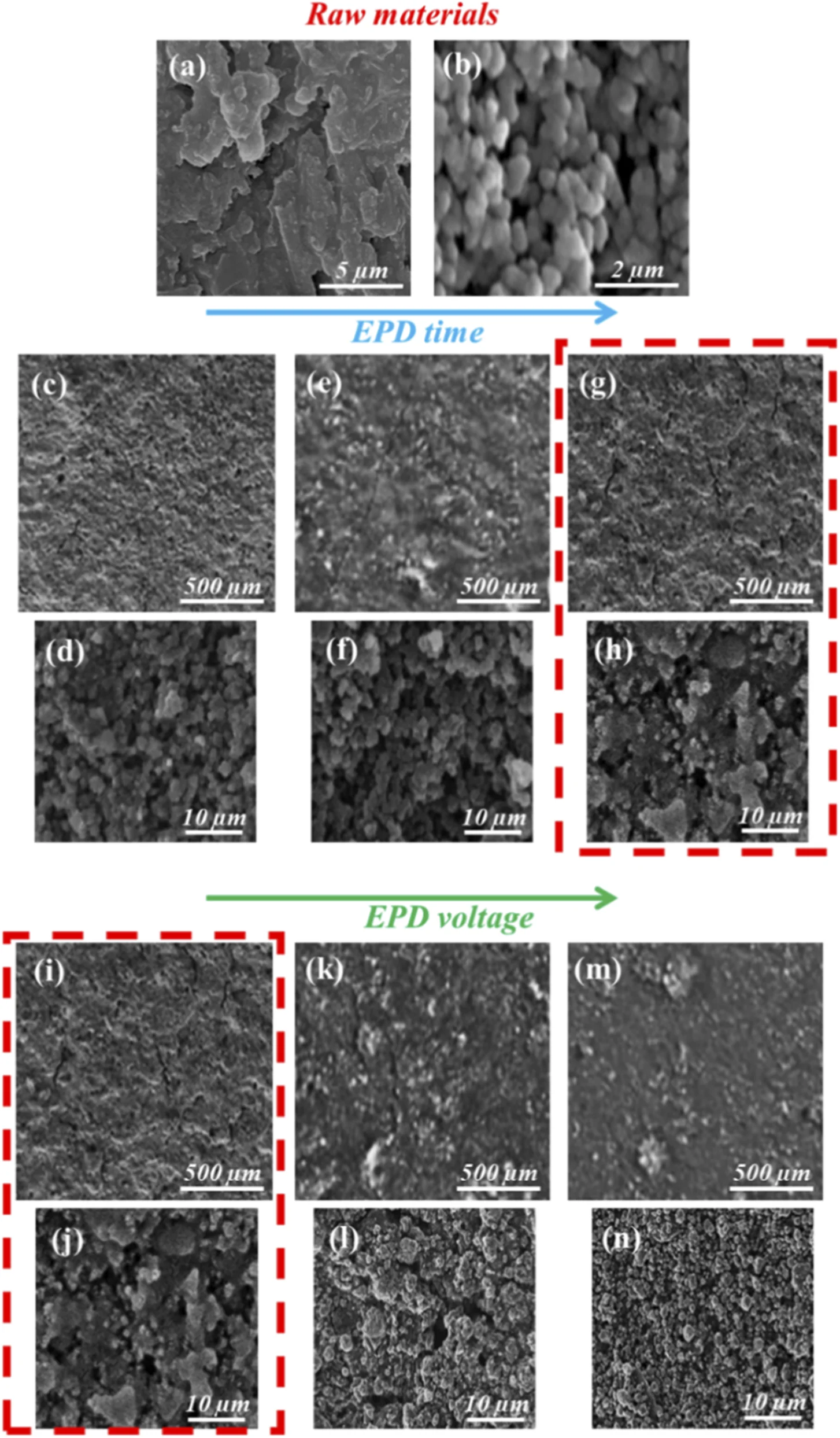
SEM images of **(a)** hydroxyapatite, **(b)** capsaicin, and deposited composite by **(c,d)** 5 min/40 V, **(e,f)** 10 min/40 V, **(g–j)** 15 min/40 V, **(k,l)** 15 min/100 V, and **(m,n)** 15 min/160 V.

Morphologies of the as-received CAP and HAP powders are presented in Figures 2a,b. The average particle sizes of HAP and CAP were 2.8 μm and 0.6 μm, respectively. The former exhibited a plate-like morphology, whereas the latter showed an irregular and fibrous morphology.

The HAP suspension exhibited a zeta potential of −28.3 mV while the HAP–CAP suspension showed a more negative value of −33.5 mV which indicates that colloidal stability was improved by CAP addition. As the zeta potential became more negative, the electrostatic repulsion among suspended particles was enhanced. This enhanced electrostatic repulsion helped maintain a homogeneous suspension and minimized particle sedimentation before deposition. Although the suspension exhibited good colloidal stability, prolonged deposition time promoted the continuous arrival and accumulation of HAP–CAP particles on the substrate surface. As a result, neighboring particles coalesced during coating growth, producing larger surface features and a relatively coarser morphology. Therefore, the observed increase in apparent particle size resulted primarily from deposition kinetics rather than instability of the suspension.

**FIGURE 3:**
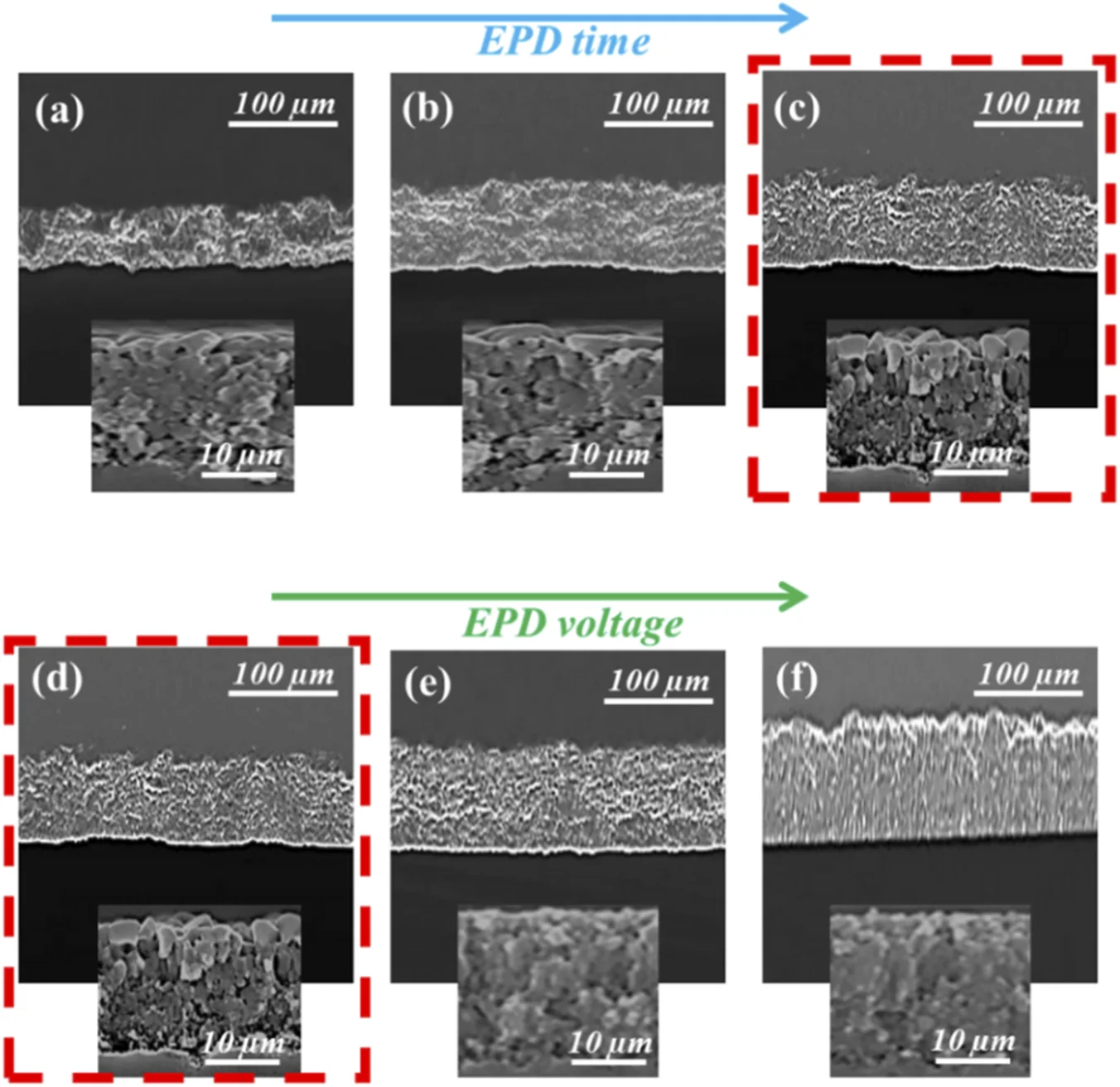
SEM images of deposited composite by **(a)** 5 min/40 V, **(b)** 10 min/40 V, **(c,d)** 15 min/40 V, **(e)** 15 min/100 V, and **(f)** 15 min/160 V.

The top-view SEM images of the coated surfaces are shown in Figures 2c–n. The porous morphology of the coatings is evident. Such interconnected porosity is advantageous because it facilitates body-fluid transport, promotes protein adsorption, and provides additional anchoring sites for cell attachment, thereby supporting subsequent tissue integration. The cross-sectional SEM images of the coated surfaces are presented in Figure 3. A continuous and relatively uniform coating structure was observed. As the EPD time increased, the coating thickness, structural uniformity, and apparent particle size increased. The increase in the apparent particle size is likely associated with particle agglomeration and the continuous accumulation of HAP–CAP particles during deposition. The observed particle agglomeration is attributed to the continuous arrival of charged particles during prolonged deposition, which increases the probability of particle–particle interaction before complete solvent evaporation. Similar findings have been reported in other biomaterials where processing conditions strongly affected the final microstructure and performance (Baihaqi et al., 2025). As the deposition time increased, neighboring particles formed larger clusters, resulting in a relatively coarser surface morphology.

The agglomeration of particles became more evident at longer EPD times. As reported by Saadati et al. (Saadati et al., 2020), short deposition times may not produce sufficiently dense coatings, whereas prolonged deposition can lead to particle accumulation and localized agglomeration. It has also been reported that increasing the EPD voltage increases the coating thickness and improves corrosion resistance (Hwang et al., 2019). Applying a higher voltage enhances the electrophoretic mobility of the suspended particles, promoting their migration and deposition onto the Mg–Ag substrate (Van Tassel and Randall, 2006). The beneficial effect of increasing EPD voltage on the uniformity of HAP coatings deposited on stainless steel has also been reported in the literature (Assadian et al., 2014). The higher electric field also increases particle flux toward the substrate, resulting in faster coating growth and reduced surface discontinuities. In the present study, increasing the deposition voltage produced more continuous and uniform coatings.

Depending on the suspension chemistry and deposition conditions, higher voltages may improve coating continuity and coverage. In general, excessively high deposition voltages may generate residual stresses during drying and increase the probability of crack formation. However, under the present experimental conditions, no severe cracking was observed. Based on these findings, EPD parameters affect coating characteristics including thickness, particle packing, and surface chemistry. Therefore, optimization of the deposition process provides an effective strategy for tailoring coating morphology, corrosion behavior, mechanical properties, and biological performance.

Figure 3 shows that the coating layer was well adhered to the substrate without obvious interfacial separation. The adhesion between the substrate and the coating is governed by mechanical interlocking and interfacial diffusion (Hiromoto and Yamazaki, 2017). The former is influenced by the initial surface roughness of the substrate, whereas the latter is enhanced by the post-deposition annealing treatment (Lin et al., 2006). It has been reported that appropriate annealing conditions improve the integrity and cohesion of HAP-based coatings (Zarbov et al., 2006). Appropriate surface roughness enhances mechanical interlocking between the coating and the substrate, thereby improving coating adhesion. However, excessively rough surfaces may act as stress concentrators and facilitate localized crack initiation (Besra and Liu, 2007; Jibril et al., 2025). The use of nano-sized HAP particles is also expected to improve coating adhesion because of their larger specific surface area, higher surface activity, and higher surface energy (Drevet et al., 2016).

**FIGURE 4:**
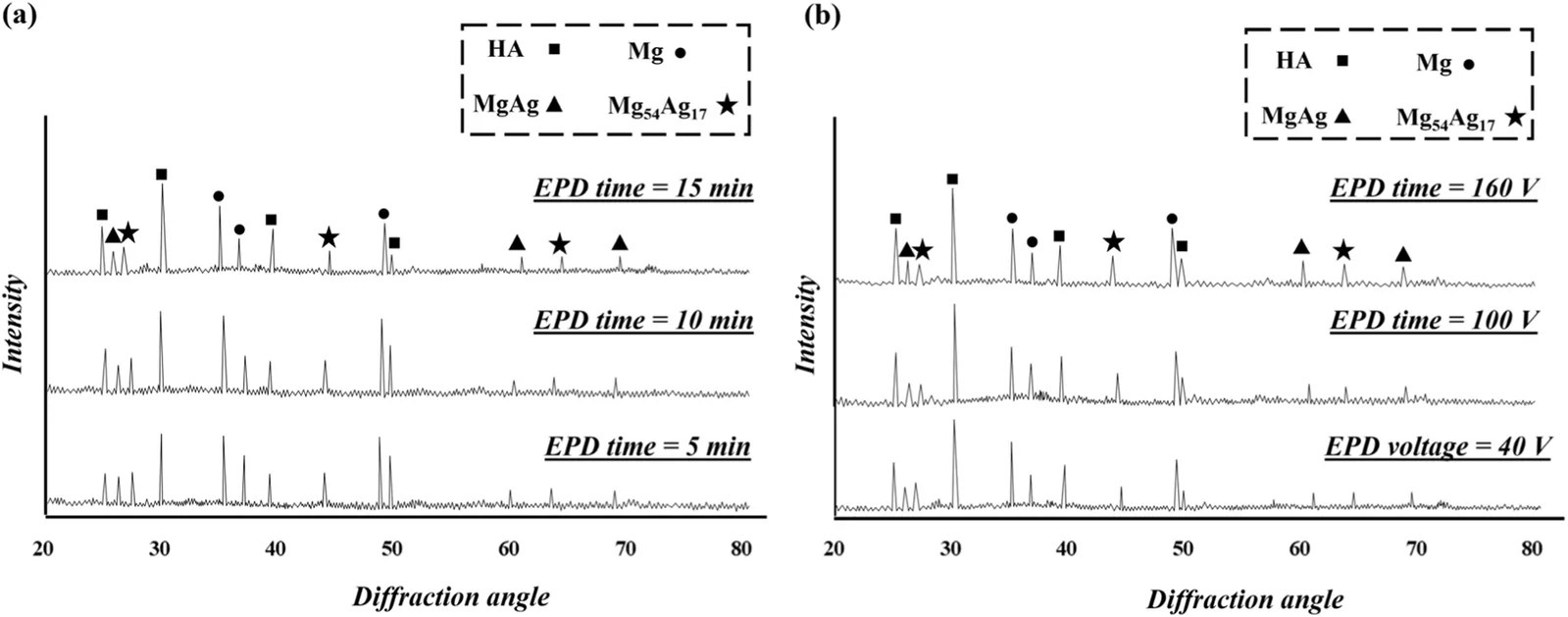
XRD patterns of deposited composite at different **(a)** EPD time and **(b)** EPD voltage.

To further investigate whether CAP incorporation and EPD parameters affected the phase constitution of the coatings, XRD analysis was performed. The XRD patterns shown in Figure 4 reveal that only the characteristic diffraction peaks of crystalline HAP were observed. No distinct diffraction peaks corresponding to CAP were detected in the HAP–CAP coatings. This observation may be attributed to the low crystallinity, amorphous nature, low concentration, overlap with HAP diffraction peaks, or homogeneous dispersion of CAP within the HAP matrix, resulting in diffraction signals below the detection limit of XRD. In addition to HAP peaks, the Mg, MgAg, and Mg_54_Ag_17_ peaks were also detected. The detection of Mg, MgAg, and Mg_54_Ag_17_ peaks indicates that the deposited coating is sufficiently thin for the X-ray beam to interact with the underlying substrate. However, as the EPD time and voltage increased, the intensity of Mg peaks decreased. This reflects the increased compactness of deposited layers at higher deposition time and voltage. The absence of crystalline CAP peaks does not indicate the absence of capsaicin within the coating. Instead, it suggests that CAP is present in an amorphous or highly dispersed state, which may facilitate more homogeneous distribution throughout the HAP matrix. Such a distribution is beneficial because it maximizes the exposure of bioactive functional groups at the coating surface without altering the crystalline structure of hydroxyapatite. Moreover, the preservation of the HAP crystalline structure indicates that CAP incorporation did not interfere with the formation or stability of the bioactive ceramic phase, which is essential for maintaining the osteoconductive properties of the coating.

**FIGURE 5:**
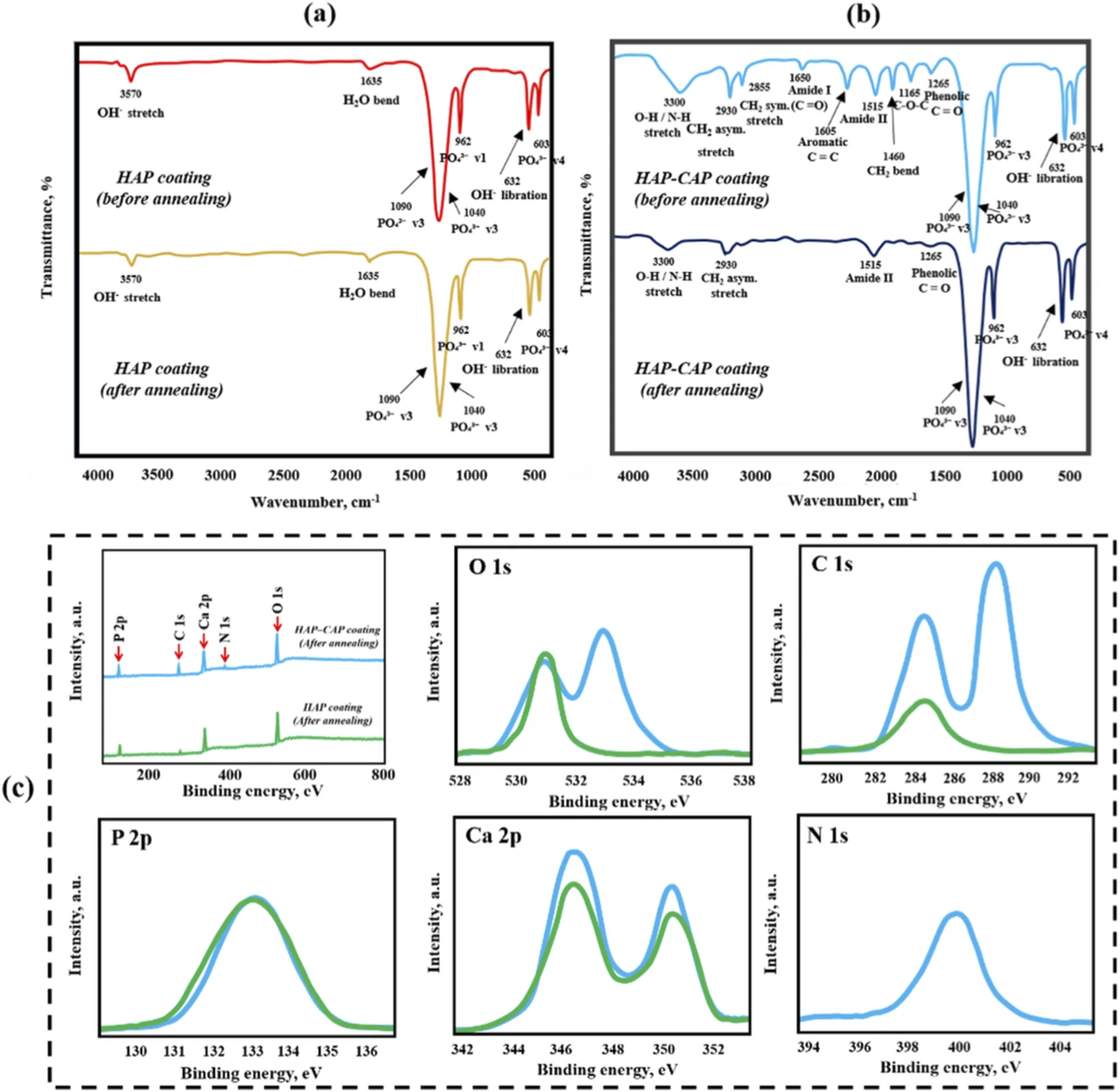
**(a,b)** FTIR spectra of the HAP and HAP–CAP coatings before and after annealing, and **(c)** XPS spectra of the annealed HAP and HAP–CAP coatings.

Figures 5a,b display the FTIR spectra of the HAP coating and HAP–CAP coating before and after annealing. The FTIR spectra of the HAP coating exhibited the characteristic absorption bands of hydroxyapatite, including the OH^−^ stretching vibration at approximately 3,570 cm^-1^, the H_2_O bending vibration near 1,635 cm^-1^, the PO_4_^3-^ ν_3_ asymmetric stretching vibrations at 1,040–1,090 cm^-1^, the PO_4_^3-^ ν_1_ symmetric stretching vibration at approximately 962 cm^-1^, the PO_4_^3-^ ν_4_ bending vibrations located at 603 and 565 cm^-1^, and the OH^−^ libration band at approximately 632 cm^-1^. The FTIR spectra show that phosphate and hydroxyl bands remained unchanged after annealing. Such features indicate that the HAP phase retained its chemical structure during heat treatment (Markovic et al., 2004).

Regarding the HAP–CAP coating before annealing, both characteristics of HAP and CAP bands were observed. The absorption bands associated with CAP include the broad O–H/N–H stretching vibration around 3,300 cm^-1^, the CH_2_ asymmetric and symmetric stretching vibrations at approximately 2,930 and 2,855 cm^-1^, respectively, the amide I (C=O) vibration near 1,650 cm^-1^, the aromatic C=C stretching vibration at approximately 1,605 cm^-1^, the amide II vibration at approximately 1,515 cm^-1^, the CH_2_ bending vibration near 1,460 cm^-1^, the phenolic C–O stretching vibration at approximately 1,265 cm^-1^, and the C–O–C stretching vibration near 1,165 cm^-1^. These organic absorption bands confirm that the incorporation of CAP into the deposited HAP coating before annealing was successful.

After annealing at 500 °C for 120 min, the characteristic HAP absorption bands remained clearly visible, thus confirming the thermal stability of the hydroxyapatite phase. In contrast, the CAP-related absorption bands became noticeably weaker but detectable. Weak O–H/N–H, CH_2_ stretching, amide I, aromatic C=C, amide II, phenolic C–O, and C–O–C absorption bands were still detected in the HAP–CAP coating after annealing which demonstrates that CAP-derived chemical functionalities were partially retained after annealing. The weakened absorption bands are attributed to partial thermal decomposition or structural modification of the organic component during annealing treatment. Compared with the HAP coating, the retained CAP-related functional groups indicate that the annealing treatment did not completely eliminate the bioactive organic component. Therefore, CAP may still contribute to the biological behavior of the coating despite partial thermal decomposition. In contrast to the HAP coating, which primarily provides osteoconductivity and corrosion protection, the retained CAP-derived functional groups may additionally contribute to the biological response through their reported anti-inflammatory and antibacterial activity. Nevertheless, the present results suggest that the improved biological performance arises from the combined effects of HAP and CAP rather than CAP alone. Therefore, the present characterization results support the conclusion that CAP acts as a complementary bioactive component to HAP rather than replacing the osteoconductive role of hydroxyapatite.

To further investigate the surface chemistry after annealing, XPS analysis was performed on the annealed HAP and HAP–CAP coatings. Figure 5c displays the survey spectra of both coatings as well as the characteristic Ca 2p, P 2p, and O 1s signals associated with hydroxyapatite (Combes and Rey, 2010). In contrast, the HAP–CAP coating exhibited a substantially stronger C 1s signal together with a distinct N 1s peak centered at approximately 399.8 eV, whereas the nitrogen peak was absent in the annealed HAP coating. Since hydroxyapatite does not contain nitrogen, the presence of the N 1s signal confirms that CAP-derived nitrogen-containing species were retained on the coating surface after annealing. The high-resolution C 1s spectrum of the annealed HAP–CAP coating exhibited a broader peak with higher intensity than that of the HAP coating. This indicates the presence of additional carbon-containing organic species derived from CAP. Furthermore, the O 1s spectrum displayed a high-binding-energy shoulder near 533 eV, which is attributed to oxygen-containing organic species originating from CAP. In contrast, the P 2p peak at approximately 133.2 eV and the Ca 2p doublet remained relatively unchanged for both coatings. This indicates that incorporation of CAP did not alter the chemical state of the hydroxyapatite phase. These XPS results further support the FTIR observations, demonstrating that hydroxyapatite remained chemically stable while a fraction of CAP-derived functional groups survived the annealing treatment. The combined FTIR and XPS results indicate that the annealing treatment preserved the structural integrity of hydroxyapatite while retaining a fraction of the CAP-derived functional groups. This balance between thermal stability and preservation of bioactive functionalities is advantageous because it allows the coating to maintain its osteoconductive characteristics while potentially providing additional biological activity through CAP incorporation. These findings further demonstrate that bioactive organic molecules can be successfully incorporated into HAP coatings while maintaining the crystalline stability of hydroxyapatite and retaining biologically relevant surface functionalities after annealing. This provides a promising strategy for developing multifunctional biodegradable implant coatings.

The retained oxygen- and nitrogen-containing functional groups originating from CAP may influence the initial interaction between the coating surface and surrounding biological fluids. Such functional groups may influence protein adsorption and subsequent cell attachment, potentially contributing to the improved biological response observed later in this study. Unlike conventional HAP coatings, the present HAP–CAP coating simultaneously preserved the crystalline structure of hydroxyapatite while retaining CAP-derived surface functionalities after annealing. To the best of our knowledge, such simultaneous preservation of crystalline hydroxyapatite and CAP-derived surface functionalities after annealing has rarely been reported for biodegradable Mg-based implant coatings. This finding provides new insight into the design of multifunctional bioactive coatings for orthopedic applications.

### 3.2 Hardness

**FIGURE 6:**
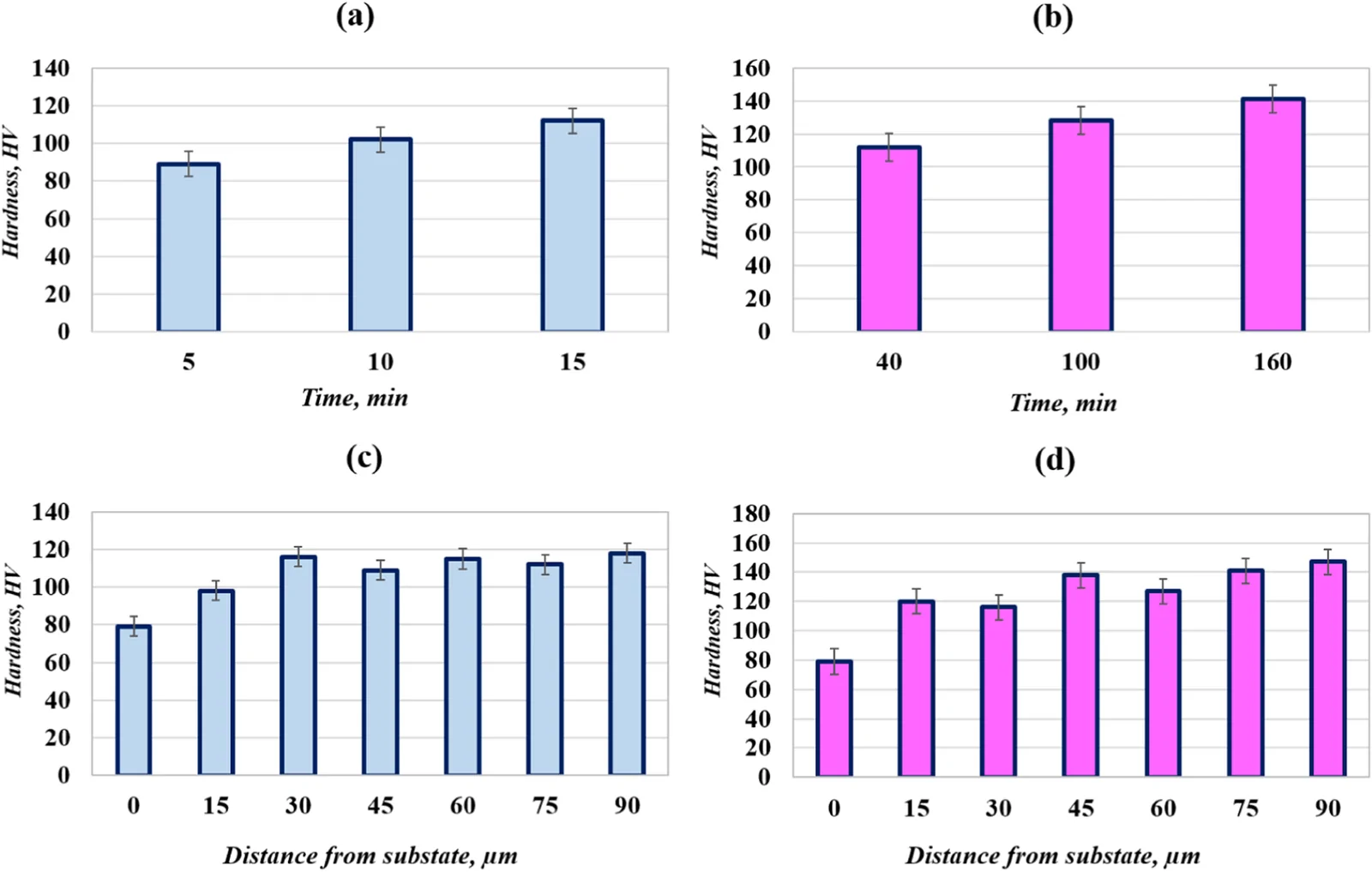
Hardness of deposited composite at different **(a)** EPD time/40 V and **(b)** EPD voltage/15 min. Hardness profile of **(c)** 15 min/40 V and **(d)** 15 min/160 V.

The hardness of the HAP–CAP coatings was measured on both the top surface and the cross-section, and the results are presented in Figures 6a,b, respectively. The hardness increased with increasing EPD voltage and deposition time. Based on microstructural observations, thicker and denser coatings were formed at higher EPD voltages and longer deposition times. Such microstructural evolution increases resistance to localized plastic deformation during indentation (Suprapto and Setyarini, 2025; Setyowulan et al., 2024). Since the HAP–CAP coating is harder than the substrate, an increase in coating thickness may dominate the indentation response of the fabricated nanocomposite. These results indicate that optimization of the EPD parameters including voltage and time improves the morphology and mechanical integrity of the deposited layer, which is important for maintaining coating durability under physiological loading conditions.

The cross-sectional hardness profiles revealed a gradual increase in hardness from the coating/substrate interface toward the outer coating surface. This hardness gradient is attributed to microstructural variations through the coating thickness. During the initial stages of electrophoretic deposition, the layers adjacent to the substrate may contain a higher density of interfacial defects, loosely packed particles, and localized porosity. As deposition proceeds, continuous particle accumulation leads to progressively denser and more compact outer layers with improved particle packing and fewer interparticle voids. Therefore, the outer region of the coating exhibits higher hardness than the inner region near the substrate. Zamharir et al. (Zamharir et al., 2021) reported that dense coating produced by the EPD process enhanced the hardness of the coating layer. The observed hardness gradient demonstrates that the coating architecture can be tailored through the EPD process, providing additional flexibility for designing coatings with location-dependent mechanical performance.

Greater fluctuations in cross-sectional hardness were observed for coatings deposited at higher EPD voltages. These fluctuations are likely associated with the higher deposition rate, which can produce local variations in particle packing density, agglomeration, and pore distribution throughout the coating thickness (Fabri et al., 2025). Despite these local variations, the overall hardness increased with increasing EPD voltage, indicating that the beneficial effects of enhanced coating density and thickness outweighed the influence of microstructural heterogeneity. These results demonstrate that controlling the EPD voltage and deposition time provides an effective approach for tailoring the mechanical performance of HAP–CAP coatings through microstructural refinement. The close agreement between the hardness measurements and SEM observations further confirms the strong relationship between coating densification and mechanical strengthening. From an application perspective, improved coating hardness is expected to enhance resistance to localized mechanical damage during implantation and early service, thereby helping to preserve coating integrity. These findings demonstrate that careful control of the EPD parameters provides an effective route for simultaneously optimizing coating microstructure and mechanical performance in multifunctional biodegradable implant coatings.

### 3.3 Corrosion properties, pH, and Mg ion release

**FIGURE 7:**
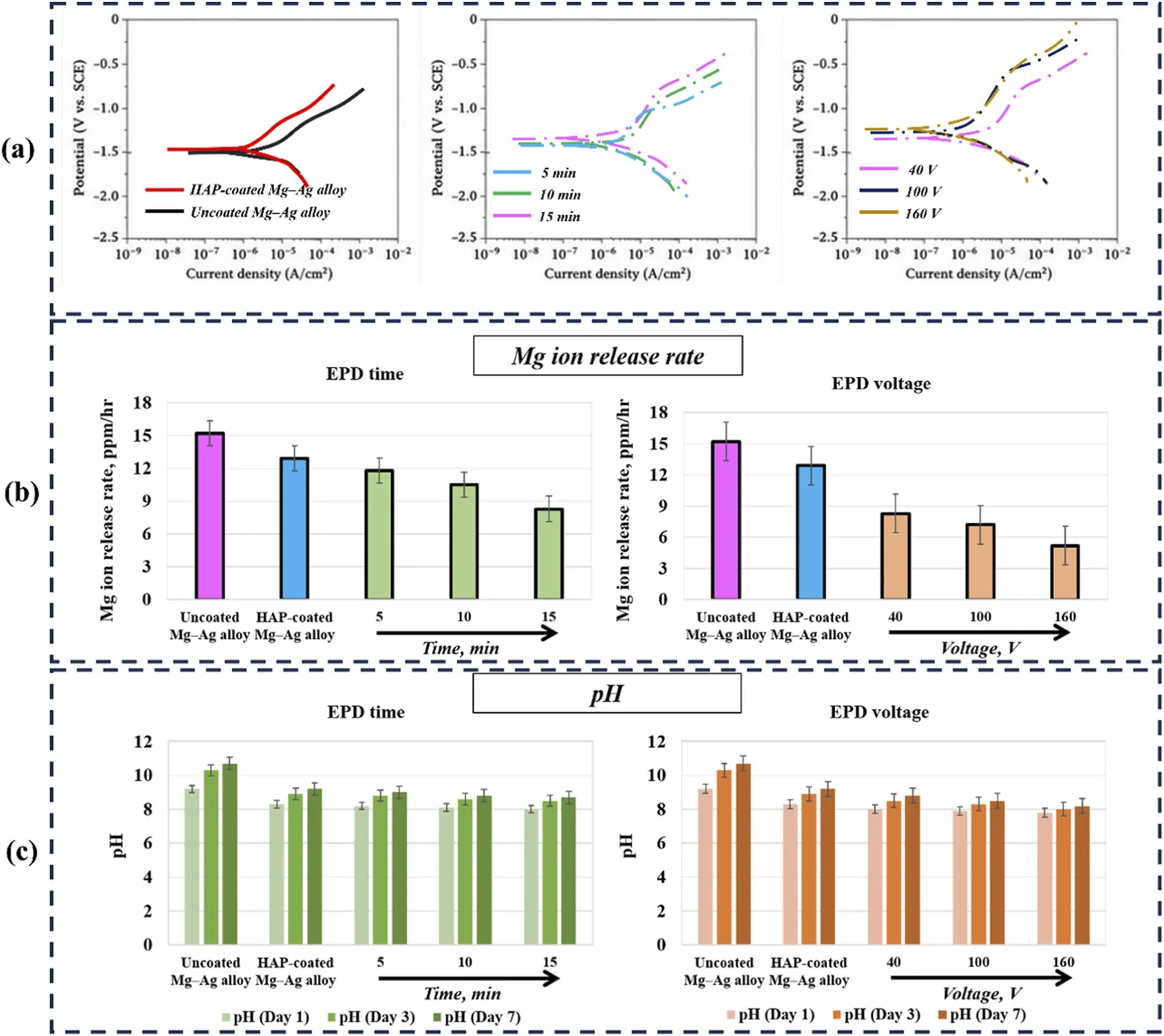
**(a)** Potentiodynamic polarization curves, **(b)** Mg^2+^ ion release, and **(c)** pH variation of the uncoated Mg–Ag alloy, HAP-coated Mg–Ag alloy, and HAP–CAP-coated Mg–Ag alloy deposited at 40 V for 5, 10, and 15 min and at 15 min using deposition voltages of 40, 100, and 160 V.

The potentiodynamic polarization curves are presented in Figure 7a. Compared with the uncoated Mg–Ag alloy, all coated samples exhibited a positive shift in the corrosion potential (E_corr_) and an evident reduction in the corrosion current density (I_corr_), thus indicating enhanced corrosion resistance. Furthermore, the HAP–CAP coatings showed superior corrosion protection compared with the HAP-coated sample, as can be seen by their more noble E_corr_ values and lower I_corr_ values. Although the present study was not designed to isolate the individual corrosion-protection mechanism of CAP, the superior performance of the HAP–CAP coatings relative to the HAP coating suggests that CAP incorporation complements the barrier effect of hydroxyapatite, resulting in enhanced corrosion protection. Increasing the EPD voltage and deposition time further decreased the corrosion current density, which is consistent with the SEM observations in Figure 2 that show an improved coating compactness and uniformity. Among all samples, the HAP–CAP coating deposited at 160 V exhibited the lowest I_corr_ value, indicating the highest resistance to electrolyte penetration and substrate degradation.

The electrochemical results are in good agreement with the Mg^2+^ ion release measurements. Samples exhibiting lower I_corr_ values also showed reduced Mg^2+^ release, confirming that the denser HAP–CAP coatings effectively suppressed the degradation of the Mg–Ag substrate. As shown in Figure 7b, the Mg^2+^ ion release decreased with increasing EPD voltage and deposition time, further confirming the improvement in corrosion resistance. The denser and more compact coatings formed under higher deposition parameters acted as an effective barrier against electrolyte penetration, thus suppressing substrate degradation and reducing Mg^2+^ ion release (Kaseem et al., 2026). The close agreement between the electrochemical measurements and Mg^2+^ release results confirms that optimization of the EPD parameters effectively improves the protective performance of the coating through microstructural refinement.

Figure 7c presents the variation in the pH of simulated body fluid (SBF) during immersion. The pH increased for all samples due to the corrosion of the Mg–Ag substrate and the associated generation of OH^−^ ions. However, the increase in pH was significantly lower for the coated samples than for the uncoated Mg–Ag alloy. Moreover, the HAP–CAP coatings deposited at higher EPD voltages and longer deposition times exhibited the smallest increase in pH. This behavior is attributed to the formation of denser and more compact coatings that effectively restrict electrolyte penetration to the substrate. The reduced degradation rate also resulted in a smaller increase in the pH of the immersion solution, demonstrating that the coating effectively mitigated excessive alkalization (Soehardjono et al., 2025; Feng et al., 2024). Hence, the degradation rate of the Mg–Ag alloy was reduced, resulting in lower alkalization of the surrounding environment. Such behavior is advantageous for biodegradable implant applications because it may provide a more physiologically favorable environment for cell attachment and proliferation (Xie et al., 2025). These findings demonstrate that controlling the coating microstructure through EPD processing not only improves corrosion resistance but also contributes to a more stable local physiological environment, which is essential for the successful performance of biodegradable orthopedic implants. Overall, the corrosion, Mg^2+^ release, and pH results consistently demonstrate that optimization of the EPD parameters substantially improves the protective performance of HAP–CAP coatings. Compared with the conventional HAP coating, incorporation of CAP provided additional improvement in corrosion performance while preserving the bioactive characteristics of hydroxyapatite. These findings demonstrate that optimization of EPD parameters together with CAP incorporation provides an effective strategy for developing multifunctional biodegradable coatings that simultaneously improve corrosion protection while maintaining the bioactive characteristics of hydroxyapatite.

### 3.4 Cell differentiation and proliferation

**FIGURE 8:**
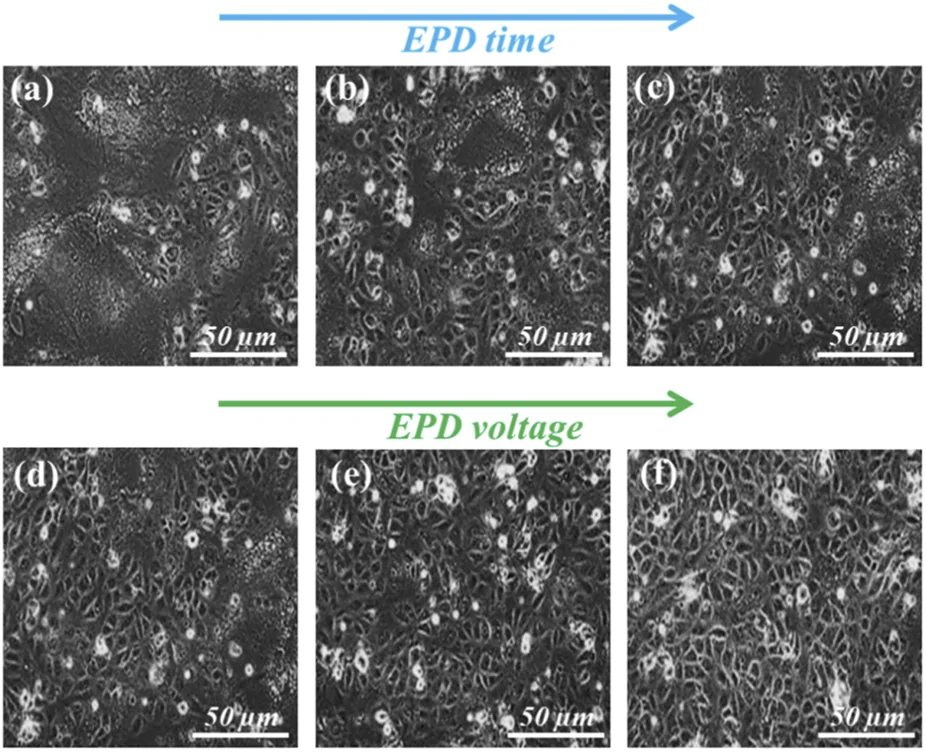
SEM images of chondrogenic differentiation of adipose-derived stem cells on deposited composite by **(a)** 5 min/40V, **(b)** 10 min/40V, **(c)** 15 min/40V, **(d)** 40 V/15 min, **(e)** 100 V/15 min, and **(f)** 160 V/15 min.

Figure 8 presents the chondrogenic differentiation of adipose-derived stem cells on deposited composite after 7 days. It can be seen that the cells possess a flattened morphology with extensive spreading and numerous filopodial extensions. Such morphology indicates strong adhesion to the coated surfaces. In addition, the cell density increased with increasing EPD voltage and deposition time. The coatings deposited at higher voltages and longer deposition times exhibited a more uniform cell distribution and greater surface coverage, suggesting enhanced cell attachment, proliferation, and chondrogenic differentiation. As reported by (Rad et al., 2014), employing the EPD method for the fabrication of HA coating on titanium substrates demonstrated higher numbers of attached and proliferated cells. These observations demonstrate that optimization of the EPD parameters promoted a coating microstructure that supported improved ADSC attachment, spreading, and chondrogenic differentiation.

**FIGURE 9:**
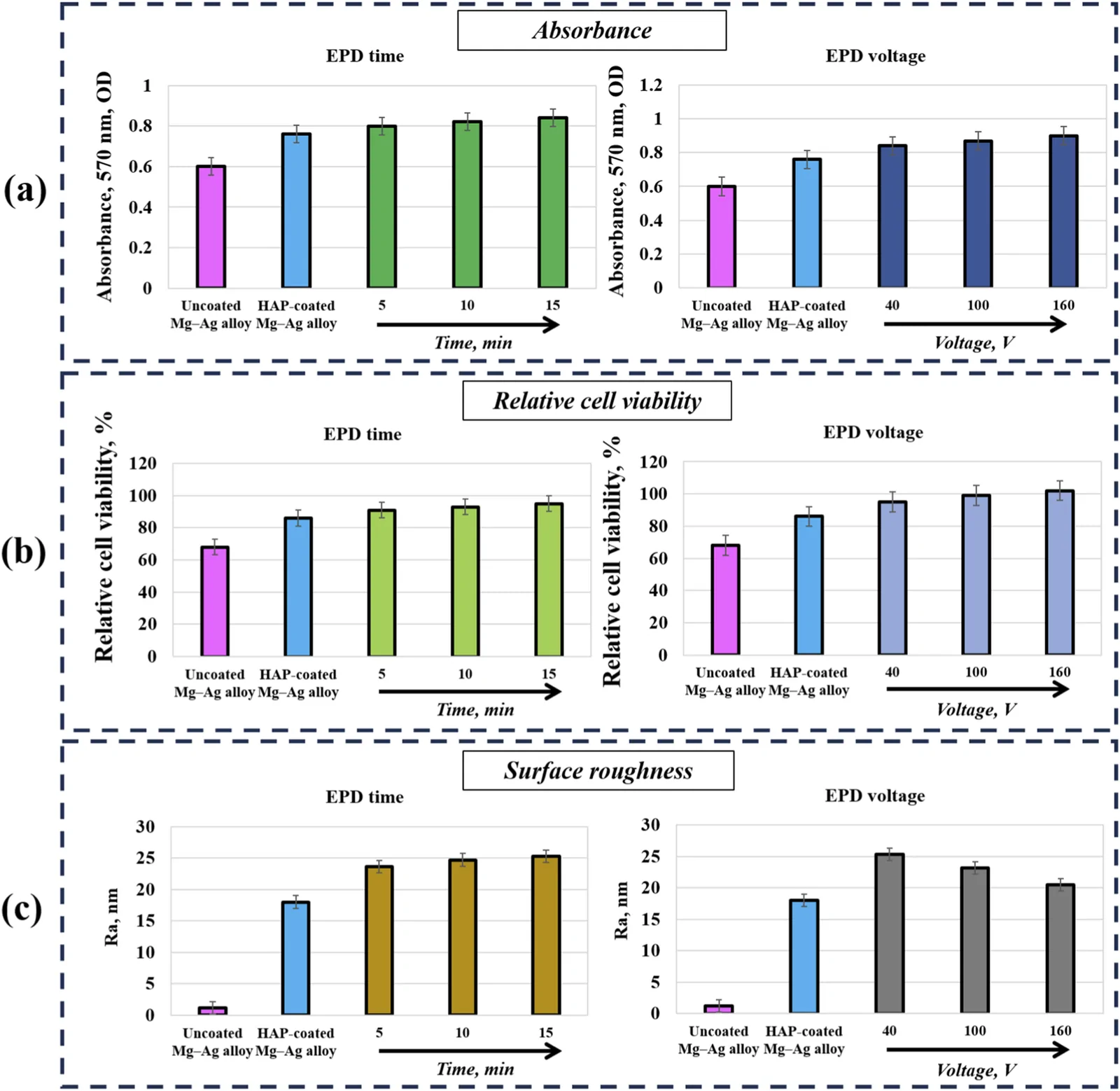
**(a)** absorbance, **(b)** relative cell viability, and **(c)** surface roughness of the uncoated Mg–Ag alloy, HAP-coated Mg–Ag alloy, and HAP–CAP-coated Mg–Ag alloy deposited at 40 V for 5, 10, and 15 min and at 15 min using deposition voltages of 40, 100, and 160 V.

The absorbance values and relative cell viability obtained from the MTT assay are presented in Figures 9a,b. The bare Mg–Ag alloy exhibited the lowest absorbance and relative cell viability, indicating poor cytocompatibility. The HAP-coated sample showed a significant improvement in cell viability compared with the uncoated alloy. It is widely accepted that the addition of HAP to the surface of Mg alloys has a positive impact on bone generation (Xu et al., 2019). In the literature (Jo et al., 2011), MgF_2_-HAP coating deposited on Mg alloy showed reduced bio-absorption of calcium phosphate due to released fluoride ions. Furthermore, the absorbance and relative cell viability increased progressively with increasing EPD voltage and deposition time for the HAP–CAP coatings. The coating deposited at 160 V for 15 min exhibited the highest absorbance and cell viability, whereas the coating deposited at 40 V for 5 min showed the lowest values among the coated samples. These findings are in good agreement with the SEM observations in Figure 8, confirming that the deposition parameters significantly influenced the biological performance of the coatings. The consistent agreement between the SEM observations and MTT results demonstrates that optimization of the coating microstructure through EPD processing directly influences the biological performance of the deposited coatings.

The enhanced cytocompatibility of the HAP–CAP coatings is closely related to their improved corrosion resistance. (Lu et al., 2012) pointed out that the cytocompatibility was improved as a result of adding HAP to the Ca-P coating on the ZK60 alloy. The electrochemical polarization results further confirmed the superior corrosion resistance of the HAP–CAP coatings through their more noble corrosion potentials and lower corrosion current densities. The improved corrosion behavior therefore provided a more stable and favorable microenvironment for ADSC growth and proliferation. As shown in Figures 7b,c, increasing the EPD voltage and deposition time resulted in lower Mg^2+^ ion release and a smaller increase in the pH of the simulated body fluid. The denser and more compact coatings formed under these deposition conditions effectively suppressed electrolyte penetration and reduced degradation of the Mg–Ag substrate.

The improved biological response can also be attributed to the bioactive nature of hydroxyapatite (Kang et al., 2022). HAP enhances cellular attachment through the release of calcium and phosphate ions (Mocanu et al., 2021). This may facilitate protein adsorption and subsequent cell adhesion. In addition, capsaicin has been reported to exhibit anti-inflammatory and antioxidant properties that may lead to favorable cellular responses (Thongin et al., 2022). Compared with the HAP coating, the HAP–CAP coating exhibited superior biological performance. Although the present study does not isolate the individual biological mechanism of CAP, the improved cytocompatibility relative to HAP alone suggests that CAP acts as a complementary bioactive component rather than replacing the osteoconductive role of hydroxyapatite. Therefore, the enhanced biological response is most likely the result of the combined effects of HAP bioactivity, CAP incorporation, and improved corrosion resistance. According to FTIR and XPS analyses in Figures 5a,b, annealing did not significantly affect the HAP phase. The FTIR and XPS results further indicate that CAP-derived surface functionalities were retained after annealing, which is consistent with the improved biological performance observed for the HAP–CAP coatings.

Surface roughness is another important factor that influences cellular behavior (Rosales-Leal et al., 2010; Corebima et al., 2025). As shown in Figure 9c, the surface roughness increased with increasing EPD time while it decreased with increasing EPD voltage. The increase in roughness with deposition time can be attributed to the continuous accumulation of HAP–CAP particles on the coating surface, whereas the reduction in roughness at higher voltages is associated with the formation of denser and more compact coatings (Salman et al., 2025). Although surface roughness exhibited opposite trends with deposition time and voltage, cell viability increased under both conditions. This indicates that surface roughness was not the dominant factor governing the biological response. Instead, the improved coating integrity, reduced Mg^2+^ ion release, lower pH variation, and enhanced corrosion resistance played a more significant role in promoting ADSC attachment and proliferation. Taken together, the biological and corrosion assessments demonstrate that the synergy between optimized EPD parameters and CAP incorporation enables the production of multifunctional HAP-based coatings with superior cytocompatibility and corrosion protection. This work advances previous investigations on conventional HAP coatings by revealing that CAP addition can enhance the biological response of resorbable Mg-based implants while retaining hydroxyapatite’s favorable bioactivity.

## 4 Conclusion

In this study, a hydroxyapatite-capsaicin nanocomposite was deposited on Mg-Ag alloy by electrophoretic deposition (EPD). The effect of different deposition voltages and times on the microstructure, surface characteristics, corrosion behavior, mechanical properties, and cell proliferation of the coated layer was examined. The main findings can be summarized as follows:

1. Based on SEM images, the deposited composites became denser and thicker at higher EPD time and voltage. However, deposition voltage was more effective in enhancing the compactness of composites. Furthermore, FTIR and XPS analyses confirmed that CAP was successfully incorporated into the HAP coating with partial retention of CAP’s surface functionalities after annealing and the sustained chemical stability of the hydroxyapatite phase. These results demonstrate that bioactive CAP can be incorporated into HAP coatings while preserving both the crystalline stability of hydroxyapatite and a portion of the CAP-derived surface functionalities after annealing.
2. The hardness of the deposited coatings increased with increasing EPD voltage and deposition time due to the formation of denser layers with lower porosity. The minimum and maximum hardness values of 89 HV and 141 HV were obtained at a deposition time of 5 min and a deposition voltage of 160 V, respectively. The hardness profile across the coating thickness showed an increasing trend from the substrate/coating interface toward the outer surface. Greater hardness fluctuations were observed at higher deposition voltages, which may be associated with local variations in particle packing and coating homogeneity.
3. The surface roughness increased with deposition time but decreased with deposition voltage. These trends were attributed to the deposition of larger particle agglomerates at longer deposition times and the formation of more compact coatings at higher voltages.
4. Increasing the EPD voltage and deposition time reduced both the Mg ion release rate and the pH increase of the SBF, indicating improved barrier performance of the HAP–CAP coating. The coating deposited at 160 V exhibited the lowest Mg ion release rate and the smallest pH variation, confirming its superior corrosion resistance and barrier performance against substrate degradation.
5. Adipose-derived stem cells exhibited improved attachment, spreading, proliferation, and chondrogenic differentiation on coatings produced at higher deposition voltages and longer deposition times. The enhanced cellular response is likely associated with the combined effects of improved corrosion resistance, reduced Mg ion release, lower pH variation, and incorporation of CAP into the HAP coating, which together produced coatings with improved cytocompatibility. Overall, this study demonstrates that electrophoretic deposition provides an effective approach for fabricating multifunctional HAP–CAP coatings on biodegradable Mg–Ag alloys. Compared with conventional HAP coatings, the incorporation of CAP improved the biological performance while preserving the structural integrity and chemical stability of hydroxyapatite. These findings provide new insight into the design of multifunctional bioactive coatings for next-generation biodegradable orthopedic implants.

## Data Availability

The raw data supporting the conclusions of this article will be made available by the authors, without undue reservation.
